# The Modulatory Effects of *Lacticaseibacillus paracasei* Strain NSMJ56 on Gut Immunity and Microbiome in Early-Age Broiler Chickens

**DOI:** 10.3390/ani12233413

**Published:** 2022-12-04

**Authors:** Sang Seok Joo, June Hyeok Yoon, Ji Young Jung, Sung Yong Joo, Su Hyun An, Byeong Cheol Ban, Changsu Kong, Myunghoo Kim

**Affiliations:** 1 Department of Animal Science, College of Natural Resources & Live Science, Pusan National University, Miryang 50463, Republic of Korea; 2 Department of Animal Science and Biotechnology, Kyungpook National University, Sangju 37224, Republic of Korea; 3 Microbial Research Department, Nakdonggang National Institute of Biological Resources (NNIBR), 137, Donam 2-gil, Sangju 37242, Republic of Korea; 4 Department of Animal Science, Kyungpook National University, Sangju 37224, Republic of Korea; 5 Research Institute of Horse Industry, Kyungpook National University, Sangju 37224, Republic of Korea; 6 Life and Industry Convergence Research Institute, Pusan National University, Mirayng 50463, Republic of Korea

**Keywords:** early-age broiler chickens, feed additive, probiotics, gut immunity, microbiome

## Abstract

**Simple Summary:**

Feed additives have been used in the livestock industry because of their positive effect on animals, including broiler chickens. Recently, probiotics, which are one of the representative feed additives for antibiotic replacement, have been widely used due to the prohibition of the use of antibiotics in the livestock industry. Various beneficial effects of probiotic *Lactobacillus* spp. have been reported; however, to our knowledge, there are few studies related to their regulating effects on gut immunity and microbiome in early-age broiler chickens. In the present study, we performed a comparative animal study on gut immunity and gut microbiome in early-age broiler chickens fed either a control diet or *Lacticaseibacillus paracasei* NSMJ56-supplemented diet. Our results suggest that *L. paracasei* NSMJ56 has modulatory effects on the gut environment, such as gut immunity and microbiome, in early-age broiler chickens.

**Abstract:**

Gut health has been attracting attention in the livestock industry as several studies suggest that it is a crucial factor for growth performance and general health status in domestic animals, including broiler chickens. Previously, antibiotics were widely used to improve livestock growth, but their use is now prohibited due to serious problems related to antibiotic resistance. Thus, finding new feed additives to replace antibiotics is drawing attention. Probiotics are representative feed additives and many beneficial effects on broiler chickens have been reported. However, many probiotic studies are focused on productivity only, and there are insufficient studies related to the gut environment, especially gut immunity and gut microbiome. In this study, we conducted an animal experiment using *Lacticaseibacillus paracasei* NSMJ56 to determine whether it has beneficial effects on gut immunity and microbiome. To evaluate the effects of NSMJ56 supplementation, newly hatched Ross 308 broiler chickens were fed an NSMJ56-containing diet for 10 days, and growth performance, antioxidant indicators, gut morphology, gut immunity-related parameters, and gut microbiome were analyzed. Flow cytometry analysis results revealed that NSMJ56 treatment increased CD4+ T cells and decreased CD8+ T cells in small intestine lamina propria and decreased *IL1b* and *IL10* gene expression in small intestine tissue. In the microbiome analysis, NSMJ56 treatment increased the alpha diversity indices and led to three enriched genera: *Massilimicrobiota*, *Anaerotignum*, and *Coprococcus*. This study suggests that NSMJ56 supplementation has regulatory effects on gut immunity and microbiome in early-age broiler chickens.

## 1. Introduction

“Gut health” has recently been recognized as a key term for domestic animals, including chickens. Gut health is determined by various biological functions of the gastrointestinal tract, including effective food digestion, microbiome diversity, and gut immune homeostasis [1]. Gut health affects not only various nutrient metabolisms but also gut immunity [2]. Although gut health may be one of the most important factors for domestic animals in terms of productivity and health status, it is more important in young animals, such as newborn chickens. This is because newly hatched chickens have not yet developed gut immunity or gut microbiome, which are both determinants of gut health [3]. For example, early-life inoculation with beneficial microorganisms directly or indirectly affects chicken health and their productivity [4,5].

As in other species of domestic animal, gut-associated lymphoid tissue (GALT) is well developed in chicken immune systems. In the gut, there are various types of GALT, including Peyer’s patches, cecal tonsils, bursa of Fabricius, and lamina propria [6]. The lamina propria, a thin vascular layer just below the gut epithelium, contains various types of immune cell, including antigen-presenting cells (APCs), B cells, and T cells [7]. These immune cells control gut tissue homeostasis through the regulation of various innate and adaptive immune responses [8]. The gut environment of animals consists mainly of epithelium, immune cells, metabolites, and microbiome. The gut environment is regulated by the interaction between the host tissue and gut microbiome [7]. Thus, gut microbiome is one of the major players in regulating gut health status. It has been reported that the commensal bacteria of chickens living on the surface of the gut mucus layer play a key role in the host metabolism and preventing infectious diseases. In addition, their metabolites, such as short-chain fatty acids, regulate gut immune cells associated with homeostatic mechanisms of gut tissue [9].

Antibiotic supplements have long been used to improve growth performance and help to control diseases in the livestock industry. However, most antibiotics potentially produce antibiotic residues in the meat or eggs of treated chickens, which has caused concerns regarding antibiotic resistance in recent years [10]. Since 2006 in Europe, the use of antibiotics as a feed additive for growth purposes has been banned in the poultry industry, and their use has been banned worldwide [11]. Therefore, it is necessary to develop new functional dietary compounds to replace antibiotics for chicken.

Feed additives as dietary ingredients can be advantageous to domestic animals by improving animal health and productivity. The most popular feed additive is probiotics such as *Lactobacillus* spp., *Bacillus* spp., or yeast [12]. As the first probiotics to be issued as a feed additive, *Lactobacillus* spp. are considered an excellent alternative to antibiotics and have been used as a feed additive owing to their safety and various positive effects on immunity, metabolism, and growth performance in broiler chickens [13]. For example, when broiler chickens were fed a probiotic diet containing *Lactobacillus* spp., growth performance indices were improved and gut immune parameters changed, including increased numbers of intraepithelial T cells and the expression of genes for Toll-like receptors (TLRs) in small intestine tissue [14]. Also, positive effects of supplementation of *Lactobacillus* spp. on gut microbiome are reported. As an example, at-hatch administration of the probiotic *Lactobacillus* strain improved weight gain via expansion of beneficial microbiota such as *Bacteroides uniformis* and *Weissella* spp. [15].

Although probiotics are attracting attention for use in the poultry industry, there are few studies on gut immunity and gut microbiome regulatory effects of *Lactobacillus* spp. (especially *L. paracasei*) in early-age broiler chickens. Therefore, in the present study, we examined the effects of *L. paracasei* strain NSMJ56 on gut immunity and microbiome of newly hatched broiler chickens. Our study suggests that NSMJ56 has potential as a gut environment-regulatory feed additive for early broiler chickens.

## 2. Materials and Methods

### 2.1. Feed Additive Prepartaion

*Lacticaseibacillus paracasei* NSMJ56 was selected for use in this study based on its high acid tolerance identified in a previous study [16]. *L*. *paracasei* NSMJ56 was cultivated in de Man, Rosaga and Sharpe (MRS) medium (BD Difco, Detroit, MI, USA) and centrifuged at 12,000 rpm for 5 min to collect a pellet. Then, the pellet was resuspended in 10% (*w/v*) skim milk (MB cell, Seoul, South Korea) as a protectant agent. The mixture was lyophilized using a freeze dryer (FD-8512, Ilshin Lab Co., Ltd., Dongducheon, Gyeonggi-do, South Korea) to obtain the powder form. The number of colony-forming units (CFU)/g of the powder sample was calculated using the standard plate count method using MRS agar (BD Difco, Detroit, MI, USA), and skim milk powder (MB cell, Seoul, South Korea) was added to achieve the final desired concentration of 5 × 10^8^ CFU/kg of viable cells.

### 2.2. Animals and Experimental Design and Sample Collection

A total of 120 newly hatched Ross 308 male broilers were obtained from a local hatchery (Samhwa breeding, Hongseong, South Korea). All birds were individually tagged with identification numbers and weighed. The broilers were assigned to two dietary treatments in a randomized complete block design with body weight as a blocking factor following the Experimental Animal Allotment Program [17]. Each dietary treatment contained 6 replicate pens with 10 birds per cage. Dietary treatments included a corn–soybean meal-based control diet and a probiotic diet prepared by adding 1 g/kg of *L. paracasei* NSMJ56 to the control diet at the expense of cornstarch (Table 1). Experimental diets were formulated to meet or exceed the recommended nutritional specifications for Ross 308 broilers [18]. All experimental diets were provided in mash form and birds had ad libitum access to water and feed throughout the experiment. Birds were housed in wire-floored battery cages (60 × 50 × 60 cm) in an environmentally controlled room with 24-hour continuous lighting. The temperature from hatching to day 4 was maintained at 33 to 34 °C and then reduced to 30 °C until day 10. Birds and feed were weighed on days 1 and 9. Dead birds were removed and weighed daily to correct the growth performance data. Experimental procedures were reviewed and approved by the Kyungpook National University Institute for Animal Care and Use Committee, Republic of Korea (KNU 2021-0213).

On day 10, one bird representing the median weight of each cage was selected and euthanized by CO_2_ asphyxiation. Blood samples were taken from the jugular vein of one bird from each cage. The jejunum tissues were then collected, and 4 cm was used for gut immune cell isolation, 1 cm for histology analysis, 0.5 cm for RNA extraction. The cecal contents were collected from the cecum of every broiler for gut microbiome analysis and volatile fatty acid (VFA) analysis. For VFA analysis, some cecal contents were pooled with other birds in the same cage.

### 2.3. Antioxidant Activity Analysis

The serum samples were obtained by centrifugation at 2000× *g* for 15 min at room temperature and stored at −20 °C prior to antioxidant activity analysis. Serum samples without dilution were used for determining the levels of malondialdehyde (MDA; Cell Biolabs, Inc., San Diego, CA, USA), catalase (CAT; Cell Biolabs, Inc., San Diego, CA, USA), glutathione peroxidase (GPx; BioAssay Systems, Hayward, CA, USA) and superoxide dismutase (SOD; BioAssay Systems, Hayward, CA, USA) as per the manufacturers’ instructions.

### 2.4. Gut Tissue Histology Analysis

Jejunum tissue was cut into 1 cm pieces and fixed in 10% neutral buffered formalin (Sigma-Aldrich, St. Louis, MO, USA). Then, tissue samples were embedded in paraffin and sectioned for hematoxylin–eosin (H&E) staining. Villus height and crypt depth were measured using ImageJ software (NIH).

### 2.5. Gut Lamina Propria Cell Isolation

Lamina propria cells (LP cells) were isolated using a slightly modified previous method [19]. Briefly, jejunum tissues were cut into 0.5 cm pieces and washed with phosphate-buffered saline (PBS; Thermo Fisher Scientific, Waltham, MA, USA) containing 1 mM DL-dithiothreitol (DTT; Sigma-Aldrich, St. Louis, MO, USA), 30 mM ethylene-diamine-tetra acetic acid (EDTA; Thermo Fisher Scientific, Waltham, MA, USA), and 10 mM 4-[2-hydroxyethyl]-1-piperazineerhanesulfonic acid (HEPES; Thermo Fisher Scientific, Waltham, WA, USA) at 37 °C for 10 min (predigestion first step). Then, tissue samples were washed again in PBS containing 30 mM EDTA and 10 mM HEPES at 37 °C for 10 min (predigestion second step). After the washing step, tissues were transferred to 5 mL of 10% fetal bovine serum (FBS) containing RPMI 1640 (GenDEPOT, Barker, TX, USA) and inverted for 2 min (neutralization step). Lastly, the tissues were digested in 10% FBS containing RPMI 1640 with 0.5 mg/ml collagenase VIII (Sigma-Aldrich, St. Louis, MO, USA) at 37 °C for 1 h (digestion step). After the digestion step, isolated cells were applied to Percoll (GE Healthcare, Chicago, IL, USA) gradient centrifugation (40% Percoll on the top, 70% Percoll on the bottom).

### 2.6. Flow Cytometry Analysis

Isolated LP cells were analyzed using FACS Canto II (BD, Franklin Lakes, NJ, USA). Dead cells were excluded using Live/Dead fixable dead cell stain (Thermo Fisher Scientific, Waltham, MA, USA). The following anti-chicken antibodies were used for staining: anti-CD3 (CT-3; SouthernBiotech, Birmingham, AL, USA), anti-CD4 (CT-4; SouthernBiotech, Birmingham, AL, USA), anti-CD8a (CT-8; SouthernBiotech, Birmingham, AL, USA), anti-TCRγδ (TCR-1; SouthernBiotech, Birmingham, AL, USA), anti-MHC II (2G11; SouthernBiotech, Birmingham, AL, USA), anti-Bu-1 (AV20; SouthernBiotech, Birmingham, AL, USA), and anti-Monocyte/Macrophage (KUL01; SouthernBiotech, Birmingham, AL, USA). All antibodies were diluted 1:200 in PBS and incubated for 30 min under dark conditions. Then, all samples were fixed using 4% paraformaldehyde (PFA) and stored at 4 °C until analysis. The analysis was conducted by two panels: (1) MHC II, Bu-1, and monocyte/macrophage for B cells and APCs; (2) CD3, CD4, CD8a, and TCR γδ for T cells.

### 2.7. RNA Extraction and qRT-PCR Analysis

The jejunum tissues were used for RNA extraction using TRIzol™ Reagent (Thermo Fisher Scientific, Waltham, MA, USA). Isolated RNA (1 ug) was used for cDNA synthesis using AccuPower^®^ RT PreMix (Bioneer, Daejeon, South Korea) in accordance with the manufacturer’s instructions. For investigation of mRNA expression level, qRT-PCR was performed using a QuantStudio 1 Real Time PCR system (Applied Biosystems, Waltham, CA, USA) with reaction conditions as follows: 50 °C for 2 min, 95 °C for 10 min, 95 °C for 20 s, and 60 °C for 40 s (40 cycles), followed by melting curve analysis. *GAPDH* was used as a housekeeping gene, and relative quantification was calculated using the 2^−ΔΔCT^ method [20]. The primer list used in this study is presented in Table 2.

### 2.8. Volatile Fatty Acid Analysis

Approximately 0.5 g of cecal content was suspended in 4.5 mL cold distilled water and mixed with 0.025 mL of saturated mercury chloride, 0.5 mL of 25% metaphosphoric acid, and 0.1 mL of 2% pivalic acid. The samples were centrifuged at 1000× *g* at 4 °C for 20 min and 1 mL of supernatant was collected and used to measure the concentration of VFA in cecal contents. The samples injected into gas chromatography (6890 Series GC System, HP, Palo Alto, CA, USA) equipped with a flame ionization detector and a capillary column (30 m × 0.25 mm × 0.25 μm; Agilent, Santa Clara, CA, USA) operated at 50 °C in the oven. The inlet and detector temperatures were 180 and 250 °C, respectively. Helium was used as a carrier gas.

### 2.9. 16S rRNA Sequencing Analysis

Bacterial DNA of cecal content samples was extracted using DNeasy PowerSoil Kit (Qiagen, Hilden, Germany). Then, the sequencing libraries were prepared according to the Illumina 16S Metagenomic Sequencing Library protocol to amplify the V3 and V4 regions. Briefly, 2 ng of DNA was amplified using universal primers and Herculase II fusion DNA polymerase (Agilent Technologies, Santa Clara, CA, USA). The PCR conditions were as follows: (1) 3 min at 95 °C; (2) 25 cycles of 30 s at 95 °C, 30 s at 55 °C, and 30 s at 72 °C; (3) 5 min at 72 °C. The universal primer set using the Illumina adapter overhang sequence used for PCR was as follows: (1) V3-F: 5′-TCGTCGGCAGGTAGAGTAGAGTAGAGCCTACGGGGCWGCAG-3′; (2) V4-R: 5′-GTCTCGGGGGGGGGTAGAGAGAGAGAGAGAGAGACHVGTATATCC-3′. The PCR products were purified with AMPure beads (Agencourt Bioscience, Beverly, MA, USA) and quantified using qPCR according to the protocol (KAPA Library Quantification kits for IlluniaSequencing platforms). Then, paired-end (2 × 300 bp) sequencing was conducted by Macrogen using the MiSeq™ platform (Illumina, San Diego, CA, USA).

After sequencing was completed, the raw data was classified for each sample using index sequences. Paired-end FASTQ files were generated for each sample and the program Cutadapt (v3.2) was used to remove adapter sequences and primer sequences in the target gene domain [21]. For the sequencing error correction, the DADA2 (v1.18.0) package, available in program R, was used [22]. Briefly, the forward and reverse sequences were cut into 250 bp and 200 bp, respectively, and sequences with expected errors of 2 or higher were excluded. Thereafter, assembling the corrected sequence and amplicon sequence variants (ASVs) were formed using the consensus method of DADA2. For the comparative analysis of microbial compositions, the normalization process was conducted using QIIME (v1.9) [23]. Each ASV sequence performed BLAST+ (v2.9.0) on the reference database to assign taxonomy information for the most similar microbes [24]. The alpha diversity analyses (Chao 1, Shannon index, Inverse Simpson) were performed using QIIME with ASV abundance and taxonomy information to confirm species diversity and uniformity of cecal content samples. Beta diversity was visualized by principal coordinates analysis (PCA) using STAMP software (v.2.1.3) [25].

### 2.10. Linear Discriminant Analysis Effect Size (LEfSe) Analysis

The linear discriminant analysis (LDA) effect size (LEfSe) analysis [26] was conducted on the website (http://huttenhower.sph.harvard.edu/galaxy) (accessed on 20 October 2022). The Kruskal–Wallis sum-rank test was used to identify significant differences in the microbiome between the two groups. Taxa with logarithmic LDA values over 2.0 were selected for the histogram figure.

### 2.11. Statistical Analysis

All experimental data are presented as means ± standard deviation (SD). Data were checked for normal distribution using the Shapiro–Wilk test in Prism 8 software (GraphPad, La Jolla, CA, USA). Then, two statistical analysis methods were used for comparisons between the two groups in this study. Abnormally distributed data were further analyzed using two-tailed Mann–Whitney tests, and normally distributed data were further analyzed using two-tailed unpaired *t*-tests. Statistical significance was taken as *p* < 0.05. The exact value of *n*, which represents the number of broilers in each experiment, is shown in each table or figure legend.

## 3. Results

### 3.1. Effect of Supplementation of NSMJ56 on Growth Performance of Early-Age Broiler Chickens

The effects of dietary *L. paracasei* NSMJ56 supplementation on the growth performance of early-age broiler chickens are shown in Table 3. Supplementation with NSMJ56 did not affect the growth performance indicators body weight gain, feed intake, or gain-to-feed ratio.

### 3.2. Effect of Supplementation of NSMJ56 on Antioxidant Activity Parameters in Sera

**Figure 1:**
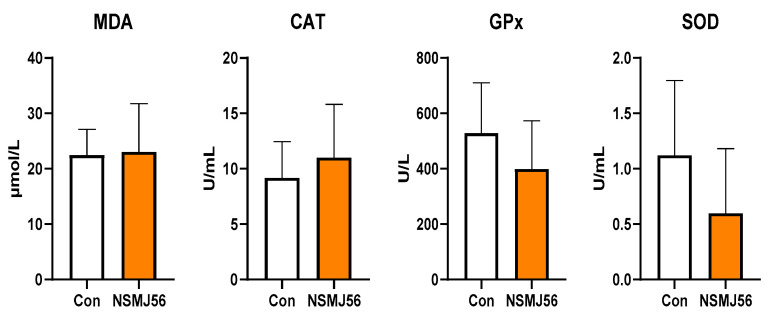
Changes in the antioxidant capacity by NSMJ56 feeding. Broilers were fed the control diet or NSMJ56-containing diet for 10 days. Total MDA and antioxidant enzyme (CAT, GPx, SOD) activity in sera were analyzed. Data were obtained from the control group (*n* = 6) and NSMJ56 group (*n* = 6) and presented as means ± SD. The significance differences between the control and NSMJ56 groups were analyzed using a two-tailed unpaired *t*-test or a Mann–Whitney test.

The different MDA and antioxidant enzyme (CAT, GPx, SOD) activities were analyzed between the control and NSMJ56 feeding groups (Figure 1). The sera were separated from the blood to confirm the antioxidant capacity of the early-age broiler chickens in the two groups. There were no significant antioxidant effects of dietary *L. paracasei* NSMJ56 supplementation.

### 3.3. Changes of Small Intestine Morphology of Early-Age Broiler Chickens by NSMJ56

The histological parameters of jejunum tissue in 10-day-old broiler chickens are presented in Table 4. In the experiment, the control and NSMJ56 groups comparisons of jejunum villus height, crypt depth, and villus height:crypt depth (VH:CD) ratio were made. However, no significant differences were found in the intestinal morphology results.

### 3.4. Changes in Immune Cells in the Small Intestinal Lamina Propria of Early-Age Broiler Chickens by NSMJ56

**Figure 2:**
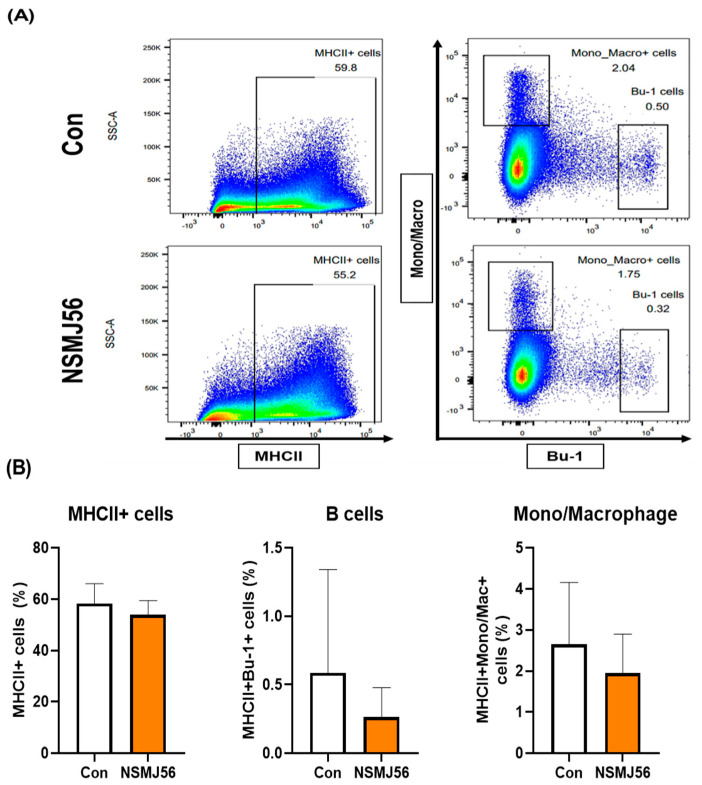
Changes in antigen-processing cells in the intestine by NSMJ56 feeding. Broilers were fed a control diet or an NSMJ56-containing diet for 10 days. Proportions of MHC II+ cells, B cells, and monocyte/macrophages were analyzed by flow cytometry in the 10-day-old broilers. A representative dot plot is shown (**A**) and immune cell distribution is presented (**B**). Data were obtained from the control group (*n* = 10) and NSMJ56 group (*n* = 7) and presented as means ± SD. Significant differences between the control and NSMJ56 groups were analyzed by a two-tailed unpaired *t*-test or Mann–Whitney test.

**Figure 3:**
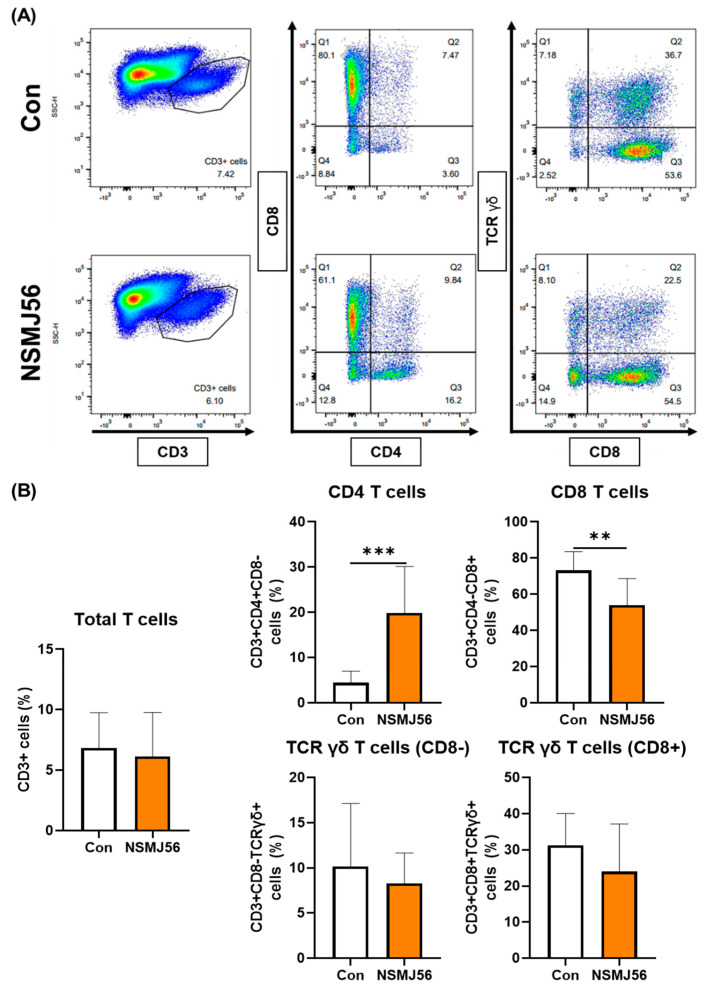
Changes in T cells in the small intestine by NSMJ56 feeding. Broilers were fed a control diet or NSMJ56-containing diet for 10 days. Proportions of total T cells, CD4 T cells, CD8 T cells, and TCR γδ T cells were analyzed by flow cytometry in the 10-day-old broilers. A representative dot plot is shown (**A**) and immune cell distribution is presented (**B**). Data were obtained from the control group (*n* = 10) and NSMJ56 group (*n* = 6) and presented as means ± SD. Significant differences between the control and NSMJ56 groups were analyzed by a two-tailed unpaired *t*-test or a Mann–Whitney test. ** *p* < 0.01 and *** *p* < 0.001.

The immune cell population of LP cells in early-age broiler chickens was assessed by flow cytometry analysis between the control and NSMJ56 diet groups. There was no significant difference in the B cells (MHC II + Bu-1+) or mono/macrophages (MHC II + Mono/Macro+), which are considered APCs, according to NSMJ56 feeding (Figure 2). However, some T cells in LP cells showed significant differences (Figure 3). We analyzed total T cells (CD3 +), CD4 T cells (CD3 + CD4 + CD8-), CD8 T cells (CD3 + CD4-CD8+), and TCR γδ T cells (CD3 + CD8- or CD8 + TCR γδ+). In our results, NSMJ56 feeding increased the population of CD4 T cells and decreased the population of CD8 T cells compared to control diet group (Figure 3).

### 3.5. Changes of Gut Immunity and Gut Health-Related Gene Expression in the Early-Age Broiler Chickens Small Intestine Tissue by NSMJ56

**Figure 4:**
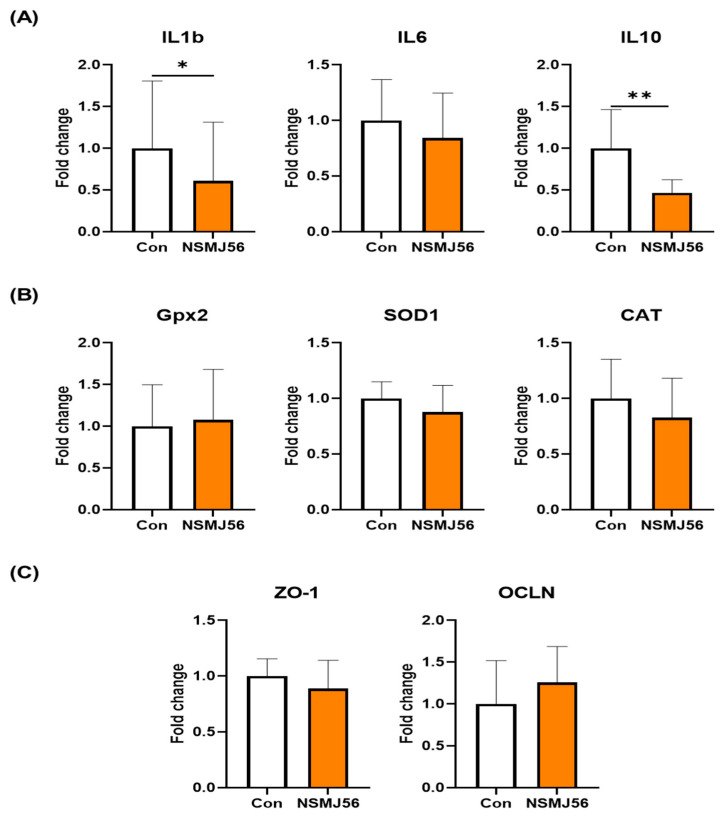
The expression of gut immunity and gut health-related genes in small intestine tissue by NSMJ56 feeding. Broiler were fed a control diet or NSMJ56-containing diet for 10 days. Cytokine genes (**A**), antioxidant enzyme genes (**B**), and tight junction protein genes (**C**) were analyzed by qRT-PCR. Data were obtained from the control group (*n* = 10) and NSMJ56 group (*n* = 8) and presented as means ± SD. Significant differences between the control and NSMJ56 groups were analyzed by a two-tailed unpaired *t*-test or Mann–Whitney test. * *p* < 0.05 and ** *p* < 0.01.

To characterize the changes in gut immunity and gut health-related gene expression following NSMJ56 feeding, qRT-PCR was conducted (Figure 4). Gene expression levels of cytokine genes (*IL1b*, *IL6*, *IL10*), antioxidant enzyme genes (*Gpx2*, *SOD1*, *CAT*), and tight junction protein genes (*ZO-1*, *OCLN*) were compared between control diet group and NSMJ56 diet group (Figure 4). The results revealed significant decreases in two cytokine genes (*IL1b*, *IL10*) in NSMJ56 diet group. However, no significant differences were observed in gut health-related genes, such as antioxidant enzymes and tight junction proteins.

### 3.6. Alteration in Cecal Content VFA of Early-Age Broiler Chickens by NSMJ56

**Figure 5:**
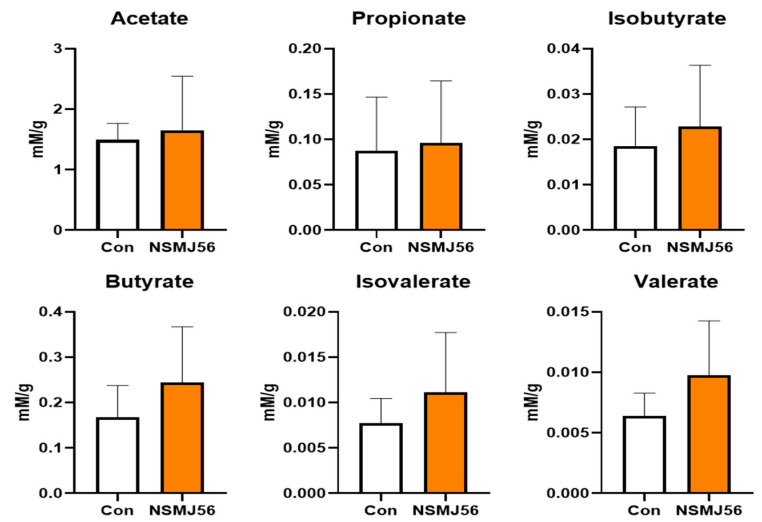
Changes in levels of cecal volatile fatty acids by NSMJ56 feeding. Broilers were fed a control diet or NSMJ56-containing diet for 10 days. The analysis was conducted to compare amounts of various volatile fatty acid production in the ceca between the control group and NSMJ56 group. Data were obtained from the control group (*n* = 6) and NSMJ56 group (*n* = 6) and presented as means ± SD. Significant differences between the control and NSMJ56 groups were analyzed by a two-tailed unpaired *t*-test or a Mann–Whitney test.

Cecal VFA analysis of early-age broiler chickens fed a control or NSMJ56 diet was conducted (Figure 5). Major VFAs, such as acetate, propionate, isobutyrate, butyrate, isovalerate, and valerate were analyzed for the control and NSMJ56 diets. Compared with the group fed the control diet, there was no significant difference from the NSMJ56-fed group.

### 3.7. Effects of NSMJ56 Feeding on the Gut Microbiome of Early-Age Broiler Chickens

**Figure 6:**
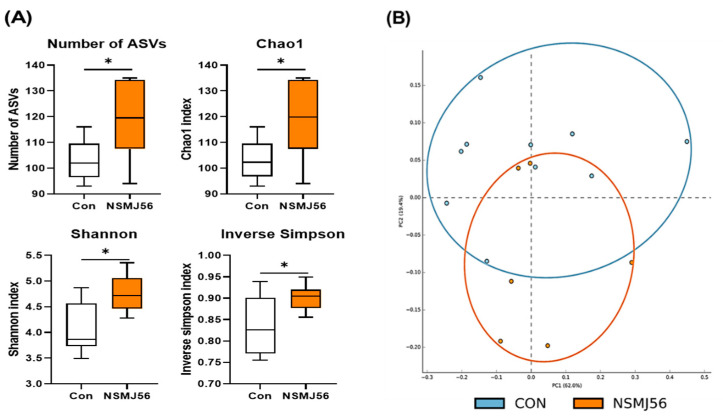
Diversity analysis of the early-age broiler gut microbiome. The alpha diversity and beta diversity of the microbiome in the ceca were compared between the control and NSMJ56 groups at the genus level. Box plots present alpha diversity indices (number of ASVs, Chao1, Shannon, and Inverse Simpson) between the two groups (**A**). Principal coordinate analysis was used for the beta diversity analysis (**B**). Alpha diversity data were obtained from the control group (*n* = 10) and NSMJ56 group (*n* = 6) and presented as means ± SD. Significant differences between the control and NSMJ56 groups were analyzed by a two-tailed unpaired *t*-test. * *p* < 0.05.

The 16S rRNA sequencing and LEfSe analysis were performed to confirm the effects of NSMJ56 feeding on the gut microbiome of early-age broiler chickens. The diversity analyses were conducted by alpha and beta diversity of broiler cecal contents (Figure 6). Alpha diversity was analyzed to determine how many different microbes were distributed in each sample. Also, beta diversity analysis was conducted to determine whether the diversity clusters of each sample were similar and to compare the differences between control diet and NSMJ56 diet groups. There were significant differences in the alpha diversity indices, such as Chao1, Shannon, and Inverse Simpson (Figure 6A). All alpha index measurements in the broiler chickens of the NSMJ56-fed group were higher than those in the control group. The beta diversity measurements are presented as a PCA plot and divided into two groups representing the two different diets. It was found that control diet and NSMJ56 diet group were partitioned by two major principal components (PC; PC1—62.0%, PC2—19.4%), respectively (Figure 6B).

**Figure 7:**
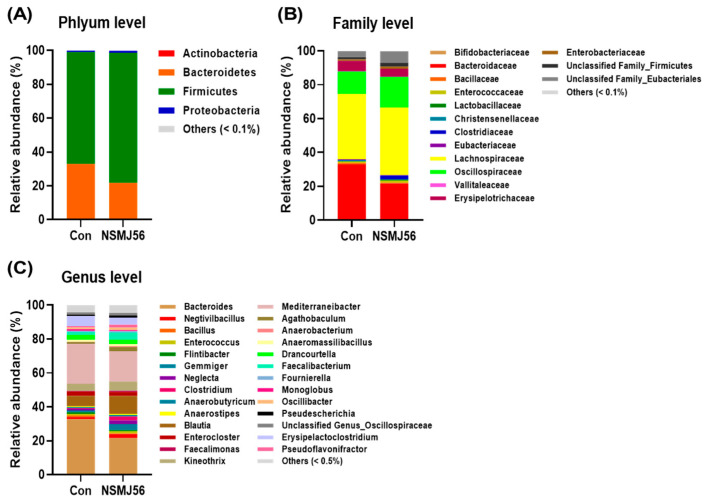
Distribution of the predominant phyla, families, and genera in the microbiome of broiler caeca. Broilers were fed with either a control diet or NSMJ56-containing diet for 10 days. 16S rRNA sequencing was conducted to determine the relative abundance at the phylum level (**A**), family level (**B**), and genus level (**C**). Proportions of the microbiome represented by less than 0.1% (phylum and family level) and less than 0.5% (genus level) were classified as “Others.”

The relative abundance of broiler gut microbiome was presented at the phylum, family, and genus levels (Figure 7). At the phylum level, *Bacteroidetes* (Con, 32.79% ± 15.64% vs. NSMJ56, 21.64% ± 12.79%), *Firmicutes* (Con, 66.06% ± 15.56% vs. NSMJ56, 76.87% ± 10.89%), and *Proteobacteria* (Con, 0.93% ± 0.99% vs. NSMJ56, 1.25% ± 2.15%) were the top three most abundant (Figure 7A). *Bacteroidaceae* (Con, 32.79% ± 15.64% vs. NSMJ56, 21.64% ± 12.79%), *Lachnospiraceae* (Con, 38.61% ± 14.15% vs. NSMJ56, 39.89% ± 11.53%), and *Oscillospiraceae* (Con, 13.15% ± 4.28% vs. NSMJ56, 18.03% ± 5.98%) were the top three most abundant families (Figure 7B), and at the genus level, the top three dominant genera were *Bacteroides* (Con, 32.79% ± 15.64% vs. NSMJ56, 21.64% ± 12.79%), *Blautia* (Con, 6.23% ± 6.89% vs. NSMJ56, 10.63% ± 7.71%), and *Mediterraneibacter* (Con, 23.37% ± 14.63% vs. NSMJ56, 17.88% ± 9.18%) (Figure 7C).

**Figure 8:**
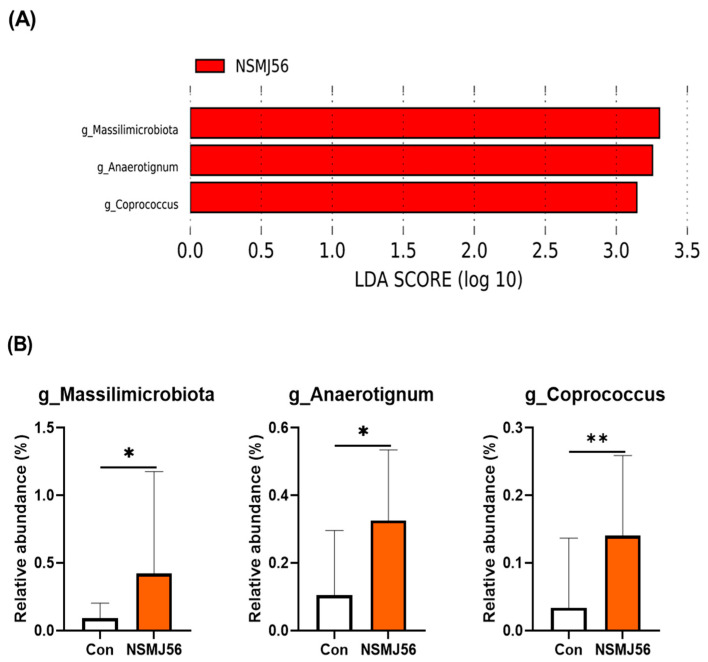
Linear discriminant analysis effect size analysis on the broiler cecal microbiome. Differentially abundant microbial distribution is shown for the control group and NSMJ56 group. A bar plot showing LDA scores of the genera that were differentially abundant in ceca microbiome of the NSMJ56 group (**A**). Microbial relative abundance of three differentially abundant genera in the NSMJ56 group were compared with the control group (**B**). Data were obtained from the control group (*n* = 10) and NSMJ56 group (*n* = 6) and are presented as means ± SD. Significant differences between the control and NSMJ56 groups were analyzed by a Mann–Whitney test. * *p* < 0.05 and ** *p <* 0.01.

For differentially abundant taxa at the phylum to genus levels between the control diet and NSMJ56 diet groups, LEfSe analysis was performed (Figure 8). There were no differentially abundant taxa in the control diet group. However, in the NSMJ56-fed broiler chicken gut microbiome, three genera were significantly increased (Figure 8A). *Massilimicrobiota*, *Anaerotignum*, and *Coprococcus* were enriched in the NSMJ56 group compared with the control group by relative abundance ratio comparison (Figure 8B).

## 4. Discussion

Gut health is a major topic not only in the field of biomedical research but also in the animal science field. In the livestock industry, gut health is important because it directly or indirectly affect productivity of domestic animals. Gut health is maintained by various components of the gut environment, such as the epithelium, immune cells, and microbiome. For example, the gut epithelium mediates barrier function to prevent from ingestion of luminal antigens. Immune cells produce various immunoregulatory molecules to fight against luminal pathogens. Gut microbiome integrates nutrient metabolism and provide defense mechanism of pathogen colonization [27]. These gut environmental factors are not independent, but they communicate with each other to perform their biological functions. Thus, dynamic interaction among gut environmental components determines gut health status. Most domestic animals, including broiler chickens, undergo dynamic changes in their gut environments at an early age. The lamina propria area contains the most immune cells (innate and adaptive) in the intestinal tissue. From the perspective of gut immune system development in the chicken, the number of innate immune cells such as mature granulocytes and heterophils is very low at hatching, but they increase 2 days after hatching and reach high numbers by day 7 after hatching [28]. The lamina propria also contains adaptive immune cells, such as B cells and T cells. These cells are immature after hatching but undergo a maturation process in the first 2 weeks of life [28]. The chicken gut microbiota also develops in the early stages after hatching. Van der Wielen et al. reported that the gut microbiome of 11-day-old broiler chickens was partially stabilized [29]. Therefore, the first 2 weeks after hatching is critical for gut health in broiler chickens, as that is when the development of the intestinal immune system and stabilization of the gut microbiome are mostly established.

Since antibiotics were discovered, they have played an important role in the development and prosperity of the livestock industry. The positive effects of dietary antibiotics have been reported, and the use of feeding antibiotics soon became a common and established practice in the livestock industry [30]. Despite these beneficial effects of antibiotics, there is a concern that the overuse of antibiotics causes antimicrobial resistance, posing a potential threat to both animal and human health. For these reasons, the use of antibiotics in the poultry industry is banned globally. Probiotics, which are antibiotic replacements, are feed additives containing live strains of microorganisms that have beneficial effects on birds. It usually improves not only the growth performance but also immune status [31]. For example, drinking water probiotic preparation containing *Enterococcus faecium* DSM 7134 and vitamins (vitamin C and vitamin D_3_) improved BW gain, gut morphology, microbial composition in the small intestine, and shown altered systemic immune status in Ross 308 broiler chickens [32]. Dietary *L. acidophilus* positively influences growth performance and gut epithelium on Kabir chickens [33]. Compared with basal diet-fed chicken, *L. acidophilus* supplemented diet-fed chickens improved BW, ADG, and feed conversion efficiency (FCE) during 42 days, which is the time point of slaughter. It also induced higher villus height, thicker mucosal layer, and more goblet cells. Unfortunately, in the present study, we did not observe a significant effect on growth performance or histological changes in intestinal tissue by NSMJ56 supplementation. We assumed that our dietary treatment is not really enough to observe positive effects of NSMJ56 on growth performance and gut tissue structural development. It would be good to try prolonged experiments with NSMJ56.

The lamina propria, one of the GALT in the chicken gastrointestinal tract, contains a variety type of immune cells, including plasma cells, effector and memory lymphocytes, macrophages, dendritic cells, and granulocytes [34]. In the present study, we examined APCs and adaptive immune cells of the jejunum of chicken. We utilized flow cytometry analysis to examine the composition of gut immune cells and successfully identified B cells, monocytes/macrophages, and T cell types. Although we did not find significant differences in APCs and B cells, NSMJ56 supplementation affected T cells especially. NSMJ56 feeding increased the population of CD4+ T cells and decreased the population of CD8+ T cells compared with control diet (Figure 3). Normally, T cells play primary roles in dynamic cellular immune responses. The CD4+ T cells stimulate phagocytosis of macrophages and antibody production by B cells, whereas CD8+ T cells directly kill the pathogen or virus-infected cells. Huang et al. reported changes in the T cells of gut tissue, such as ileum, cecum, and rectum, according to probiotic treatment in broiler chickens [35]. They used three probiotic species (*Streptococcus faecalis*, *Clostridium buthricum*, *B. mesentericus*) for one-day-old chunky broiler chickens. These probiotics caused a significant influx in the CD8+ T cells, not CD4+ T cells. In other probiotic feeding research, Asgari et al. reported that lactic acid bacteria probiotics can affect the distribution of lymphocyte subpopulations in the mucosal tract of young chickens [36]. They fed *L. acidophilus* to newly hatched Ross 308 chickens for 21 days. In the ileum immunohistochemistry results, probiotics induced upregulation of CD4+ T cell percentage; however, there was no effect on CD8+ T cells. In general, change in gut immunity, especially immune cell composition, in the gut of domestic animals with aging is unknown. In our results, we could not elucidate how NSMJ56 strain specifically induce changes in T cell subsets, CD4+ T cell and CD8+ T cells, or what the functional role of these changes induced by NSMJ56 treatment were. Therefore, in future research of early-stage broiler chickens, studies on the distribution of gut immune cells according to probiotic characteristics and related mechanisms should be conducted.

It has been reported that probiotics have immune-regulatory effects such as modulation of the gut immune cell population and activation of various immune cell in the gut [37]. They stimulate the production of immune-related molecules, such as mucins, defensins, chemokines, and cytokines in livestock animals, including chickens [38]. Cytokines are proteins or peptides secreted by various cell types which play a crucial role in immune homeostasis, such as inflammatory response [39]. In this study, NSMJ56 supplementation to early-age broiler chickens downregulated cytokine genes for *IL1b*, in jejunum tissue (Figure 4A). IL-1β is commonly referred to as a proinflammatory cytokine and is produced as part of the induce innated response [40]. There are some interesting reports that probiotics reduced expression pro-inflammatory cytokines [41]. Zheng et al. reported that probiotics alleviated proinflammatory cytokine genes on heat-stressed broiler chickens. The proinflammatory cytokine genes, such as *IL1b*, *IL6,* and *TNF-* α were increased by heat stress, but probiotic cocktails which are contained *Bacillus* spp. and *Clostridium butyricum* induced downregulation of cytokine genes, such as *IL1b* and *IL6*, in jejunum mucosa. Although we were missed on experimental design that would challenge immunity in our study, the regulation of *IL1b* gene expression by NMSJ56 supplementation in early-age broiler chicken gut tissue may have certain implication on inflammatory responses.

Chicken guts are naturally colonized with dynamic microorganisms when hatched, and specific microbes settle in various regions, such as the gizzard, small intestine (duodenum, jejunum, ileum), large intestine (colon, rectum), and ceca [42]. The chicken ceca are part of the hindgut, contain the highest density of microbes, and where fermentation of indigestible carbohydrates occurs [43]. The major cecal microbiome has been reported at the genus (*Lactobacillus*, *Ruminococcus*) and family (*Clostridiaceae*, *Lachnospiratceae*, *Runinococcaceae*, *Enterococcaceae*, *Enterobacteriaceae*, and *Bacteroidaceae)* levels [44,45,46]. In the present study, NSMJ56 increased alpha diversity of the gut microbiome in the early-age broiler chicken cecal contents, compared with control diet feeding (Figure 6). Diversity of the gut microbiome is emerging as a critical determinant of host health, and loss of microbe diversity has been related to poor gut health and immunity [47]. In general, higher microbial diversity is commonly associated with a healthier bird status, and lack of sufficient microbial diversity can affect the growth of birds [48]. For example, exposing early-age chicks to the mature microbe which are secured from healthy adult chickens increased the rate of stable microbial development at a younger age [49]. Therefore, the increase in gut microbiome diversity in the NSMJ56-fed early-age broiler chickens may have beneficial effects, and it needs to study in a future study. In addition, to understand the mechanism of gut microbiome regulation by specific microbe strain, various experimental techniques (in vitro or ex vivo studies) and observations at specific timepoints of growing birds will be required in future studies.

Through the LEfSe analysis, we identified differentially abundant taxa at the genus levels of the NSMJ56 group (Figure 8). Three genera, *Massilimicrobiota*, *Anaerotignum*, and *Coprococcus*, were enriched in the NSMJ56 diet group compared with the control diet group. Not much is known about *Massilimicrobiota*; however, some studies have been conducted on *Anaerotignum* and *Coprococcus* in the chickens [50,51]. Zenner et al. reported that synthetic cultured chicken gut microbes can influence the early-life immune system of chickens [50]. A total of nine species, including *A. lactatifermentans*, were selected by comparison between specific pathogen-free (SPF) birds and maternal microbiota exposed birds. The nine species of microbes isolated and cultured from adult chicken gut microbes were inoculated into hatched chicks, and their plasma IgA was significantly higher than that of the group treated with PBS at the age of 25 days. In our 16S rRNA sequencing data, there were two *Anaerotignum* species (*A. aminivorans*: Con, 0.03% vs. NSMJ56, 0.17%; *A. lactatifermentans*: Con, 0.08% vs. NSMJ56, 0.15%). *Anaerotignum* genera are known short-chain fatty acid producers [52]. Another NSMJ56 enriched genus, *Coprococcus,* was studied with *S. enterica* inoculation to chickens [51]. *Coprococcus* played major roles in protecting against *S. enterica* at an early stage (before 7 days postinoculation), and showed positive correlations between well-known beneficial microbes, such as *Bacillus* and *Blautia*.

In summary, this study has determined the effects of NSMJ56 as a feed additive on gut environments such as gut immunity and microbiome of early-age (first 10 days) broiler chickens. The NSMJ56 regulates T cell population and cytokine gene expression in the small intestine. Additionally, it increased gut microbial diversity and enriched potentially beneficial bacterial taxa such as *Anaerotignum*, and *Coprococcus*. However, since the present experimental design did not contain an environment- or immune-challenging condition, we missed dynamic changes of immune parameters and microbiome in the gut. We could not observe positive effect on growth performance and tissue homeostasis may be because we treated animals for a short time only. Therefore, further studies should be conducted adapting a challenge model and prolonged experiment period. It is necessary to understand the regulatory mechanism of gut immunity and gut microbiome of NSMJ56 in early-age broiler chickens.

## 5. Conclusions

In this study, we conducted an experiment to determine the effects of *L. paracasei* NSMJ56 strain on gut immunity and microbiome of early-age (first 10 days) broiler chickens. The supplementation of NSMJ56 increased CD4+ T cells in the small intestinal lamina propria and gut microbial diversity with potentially beneficial microbe genera—*Anaerotignum* and *Coprococcus*. However, 10 days of probiotic treatment did not affect growth performance, antioxidant capacity, or gut morphology. Through this study, we found potential for NMSJ56 to regulate gut environments in the early-age broiler chicken. It suggests the possibility of using NSMJ56 as an alternative feed additive to improve gut health in the poultry industry.

## Figures and Tables

**Table 1:** Ingredients and chemical compositions of experimental diets (as-fed basis).

Items	Experimental Diets ^(1)^	
	Con	NSMJ56
Ingredient composition, g/kg		
Corn	535.44	535.44
Soybean meal	388.00	388.00
Cornstarch	5.00	4.00
*L. paracasei* (NSMJ56)	-	1.00
Soybean oil	20.00	20.00
L-Arg	0.93	0.93
L-His	0.29	0.29
L-Ile	1.00	1.00
L-Lys-HCL	3.49	3.49
L-Met	2.35	2.35
L-Cys	1.43	1.43
L-Thr	1.43	1.43
L-Val	2.01	2.01
Limestone	10.58	10.58
Dicalcium phosphate	19.05	19.05
Salt	4.00	4.00
Vitamin premix ^(2)^	2.00	2.00
Mineral premix ^(3)^	2.00	2.00
Choline chloride	1.00	1.00
Calculated chemical compositions		
Nitrogen-corrected metabolizable energy, kcal/kg	2976	2972
Crude protein, %	23.00	23.00
Calcium, %	0.96	0.96
Non-phytate phosphorus, %	0.48	0.48
Calculated amino acids compositions, g/kg		
SID Arg	13.12	13.12
SID His	4.74	4.74
SID Ile	8.71	8.71
SID Leu	15.25	15.25
SID Lys	12.80	12.80
SID Met	4.75	4.75
SID Cys	3.96	3.96
SID Phe	9.17	9.17
SID Thr	8.13	8.13
SID Trp	2.28	2.28
SID Val	10.12	10.12

SID, standardized ileal digestible; ^(1)^ experimental diets were formulated by adding NSMJ56 into a control diet to reach 1 g/kg of diet at the expense of cornstarch; ^(2)^ vitamin premix supplied per kilogram of diet: vitamin A, 24,000 IU; vitamin D_3_, 8000 IU; vitamin E, 160 mg; vitamin K_3_, 8 mg; vitamin B_1_, 8 mg; vitamin B_2_, 20 mg; vitamin B_6_, 12 mg; pantothenic acid, 40 mg; folic acid, 4 mg; niacin, 12 mg; ^(3)^ mineral premix supplied per kg of diet: iron, 120 mg; copper, 320 mg; zinc, 200 mg; manganese, 240 mg; cobalt, 2 mg; selenium, 0.6 mg; iodine, 2.5 mg

**Table 2:** List of chicken primers used for this study.

Gene	Forward	Reverse	Product Size (bp) ^(1)^
*IL1b*	GCTCTACATGTCGTGTGTGATGAG	TGTCGATGTCCCGCATGA	80
*IL6*	CTCCTCGCCAATCTGAAGTC	GGATTGTGCCCGAACTAAAA	164
*IL10*	CATGCTGCTGGGCCTGAA	CGTCTCCTTGATCTGCTTGATG	94
*Gpx2*	ACGGCACCAACGAGGAGAT	TTCAGGTAGGCGAAGACGG	133
*SOD1*	AGGGGGTCATCCACTTCC	CCCATTTGTGTTGTCTCCAA	122
*CAT*	TTACGGAGGTAGAACAGATGG	TGTCAGGATACGCAAAGAGA	105
*ZO-1*	GCCAACTGATGCTGAACCAA	GGGAGAGACAGGACAGGACT	141
*OCLN*	GATGGACAGCATCAACGACC	CTTGCTTTGGTAGTCTGGGC	142
*GAPDH*	GTCCTCTCTGGCAAAGTCCAAG	TCACAAGTTTCCCGTTCTCAGC	139

^(1)^ bp; base pair.

**Table 3:** Effects of *L. paracasei* NSMJ56 on growth performance in early-age broiler chickens ^(1)^.

Item	Experimental Diets ^(2)^		
	Con	NSMJ56	*p*-Value ^(3)^
Body weight gain, g/bird	156.20 ± 8.56	152.50 ± 16.02	0.6324
Feed intake, g/bird	175.00 ± 9.69	169.00 ± 11.32	0.3466
Gain to feed ratio, g/g	0.89 ± 0.03	0.90 ± 0.06	0.7519

^(1)^ Data were obtained from Con (*n* = 6) and NSMJ56 (*n* = 6) and presented as means ± SD; ^(2)^ Con = a basal diet group, NSMJ56 = *L. paracasei* NSMJ56 diet group; diets were formulated by adding *L. paracasei* NSMJ56 into a control diet to reach 1 g/kg of diet at the expense of cornstarch; ^(3)^ significance between control and NSMJ56 group was analyzed by two-tailed unpaired *t*-test.

**Table 4:** Effects of *L. paracasei* NSMJ56 on small intestine morphology in early-age broiler chickens ^(1)^.

Item	Experimental Diets ^(2)^		
	Con	NSMJ56	*p*-Value ^(3)^
Villus height, µm	469.56 ± 33.19	459.86 ± 96.84	0.1797
Crypt depth, µm	107.13 ± 22.66	96.29 ± 7.44	0.2915
Villus height:crypt depth	4.53 ± 0.89	4.74 ± 0.61	0.6442

^(1)^ Data were obtained from Con (*n* = 6) and NSMJ56 (*n* = 6) groups and presented as means ± SD; ^(2)^ Con = a basal diet control group, NSMJ56 = *L. paracasei* NSMJ56 diet group; diets were formulated by adding *L. paracasei* NSMJ56 into a control diet to reach 1 g/kg of diet at the expense of cornstarch; ^(3)^ significant differences between the control and NSMJ56 groups were analyzed using a two-tailed unpaired *t*-test or a Mann–Whitney test.

## Data Availability

Not applicable.
